# 3-(4-Chloro­phen­yl)-1-(4-nitro­phen­yl)benzo[*f*]quinoline

**DOI:** 10.1107/S1600536809031122

**Published:** 2009-08-12

**Authors:** Shu-Liang Wang, Qing Li, Xiang-Shan Wang, Shu-Jiang Tu

**Affiliations:** a School of Chemistry and Chemical Engineering, Xuzhou Normal University, Xuzhou Jiangsu 221116, People’s Republic of China

## Abstract

In the title compound, C_25_H_15_ClN_2_O_2_, the pyridine ring is inclined at angles of 6.89 (7), 4.24 (9) and 66.98 (4)° with respect to the naphthalene, chloro­phenyl and nitro­phenyl rings, respectively. The two substituent aromatic rings make a dihedral angle of 71.1 (1)° with one another. C—H⋯π and π–π stacking are present in the crystal structure; the π–π stacking [centroid–centroid distance between the pyridyl rings of adjacent mol­ecules= 3.7838 (11) Å] links the mol­ecules into dimers, while the C—H⋯*Cg* type π–ring inter­actons link the mol­ecules into a chain structure along *c*.

## Related literature

Quinoline and its derivatives are inter­mediates in organic synthesis and are useful dyes, see: Brock *et al.* (1999 ▶). They possess a broad spectrum of biological activity, such as anti­asthmatic, anti­inflammatory and anti­malarial, see: Fokialakis *et al.* (2002 ▶); Ma *et al.* (2004 ▶); Sawada *et al.* (2004 ▶). In addition, quinoline derivatives have been evaluated as anti­cancer and anthelmintic agents, see: Sakata *et al.* (1988 ▶). For related structures, see: Tu *et al.* (2006 ▶); Xie *et al.* (2009 ▶).
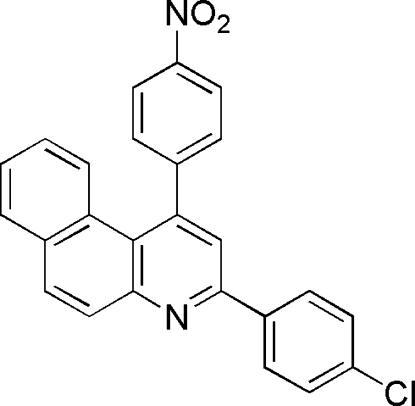


## Experimental

### 

#### Crystal data

C_25_H_15_ClN_2_O_2_
*M* *_r_* = 410.84
Triclinic, 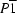

*a* = 9.1390 (12) Å
*b* = 9.5350 (11) Å
*c* = 11.9668 (17) Å
α = 108.182 (4)°
β = 105.366 (4)°
γ = 92.739 (3)°
*V* = 945.6 (2) Å^3^
*Z* = 2
Mo *K*α radiation
μ = 0.23 mm^−1^
*T* = 113 K
0.34 × 0.32 × 0.22 mm

#### Data collection

Rigaku Saturn diffractometer
Absorption correction: multi-scan (*CrystalClear*; Rigaku, 1999 ▶) *T*_min_ = 0.926, *T*_max_ = 0.952
11798 measured reflections
4467 independent reflections
3779 reflections with *I* > 2σ(*I*)
*R*_int_ = 0.034

#### Refinement

*R*[*F* ^2^ > 2σ(*F* ^2^)] = 0.049
*wR*(*F* ^2^) = 0.129
*S* = 1.06
4467 reflections
271 parameters
H-atom parameters constrained
Δρ_max_ = 0.36 e Å^−3^
Δρ_min_ = −0.47 e Å^−3^

### 

Data collection: *CrystalClear* (Rigaku, 1999 ▶); cell refinement: *CrystalClear*; data reduction: *CrystalClear*; program(s) used to solve structure: *SHELXS97* (Sheldrick, 2008 ▶); program(s) used to refine structure: *SHELXL97* (Sheldrick, 2008 ▶); molecular graphics: *SHELXTL* (Sheldrick, 2008 ▶); software used to prepare material for publication: *CrystalStructure*/MSC (Rigaku/MSC (2003 ▶).

## Supplementary Material

Crystal structure: contains datablocks global, I. DOI: 10.1107/S1600536809031122/pv2192sup1.cif

Structure factors: contains datablocks I. DOI: 10.1107/S1600536809031122/pv2192Isup2.hkl

Additional supplementary materials:  crystallographic information; 3D view; checkCIF report

## Figures and Tables

**Table 1:** Hydrogen-bond geometry (Å, °)

*D*—H⋯*A*	*D*—H	H⋯*A*	*D*⋯*A*	*D*—H⋯*A*
C24—H24⋯*Cg*^i^	0.95	2.72	3.510 (12)	142

Symmetry code: (i) 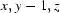. *Cg* is the centroid of the C14–C19 ring.

**Fig. 1.**
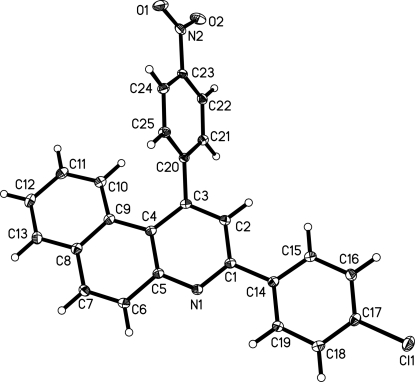
The molecular structure of (I) plotted with 50% probability of displacement ellipsoids and the atom-numbering scheme.

**Fig. 2.**
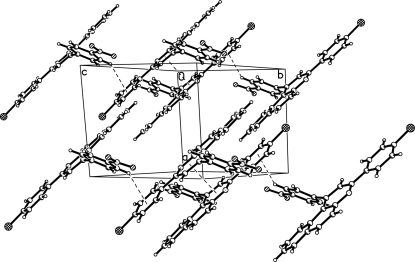
The molecular packing diagram showing the C—H···π and π–π stacking in the crystal of (I).

## supplementary crystallographic information

### Comment

Quinoline and its derivatives represent an important class of nitrogen-containing heterocycles as they constitute useful intermediates in organic synthesis and are useful dyes (Brock *et al.,* 1999). They are well known in the pharmaceutical industry and have been shown to possess a broad spectrum of biological activities including such as antiasthmatic, antiinflammatory and antimalarial activity (Sawada, *et al.,* 2004; Ma *et al.,* 2004; Fokialakis *et al.,* 2002). In addition, quinoline derivatives have been evaluated as anticancer and anthelmintic agents (Sakata *et al.,* 1988). We report here the preparation and crystal structure of 3-(4-chlorophenyl)-1-(4-nitrophenyl)benzo[*f*]quinoline, (I).

In the structure of (I) (Fig. 1), the benzoquinoline moiety is not quite planar as C3 deviates by 0.169 (1) Å from the mean-plane formed by the atoms N1/C1–C13. The pyridine ring is inclined at angles 6.89 (7), 4.24 (9) and 66.98 (4) ° with respect to the naphthalene (C4–C13), and phenyl rings C14–C19 and C20–C25, respectively. The two phenyl rings make a dihedral angle of 71.1 (1) °.

The C—H···π and π–π stacking are present in the crystal structure of (I). The π–π stacking (Cg···Cg distance 3.7838 (11) Å between the pyridyl rings of adjacent molecules) links the molecules into dimmers, while the C24—H24A···*Cg* stacking links the molecules into polymers (Figure 2).

The following crystal structures of compounds closely related to (I) have been reported: 13-(4-fluorophenyl)-12H-benzo[f]indeno[1,2-b]quinolin-12-one (Tu *et al.,* 2006) and 5-(4-bromophenyl)-1,2,3,4-tetrahydrobenzo[a]phenanthridine (Xie *et al.,* 2009).

### Experimental

The title compound, (I), was prepared by the reaction of 4-chlorobenzaldehyde (2 mmol, 0.281 g), naphthalen-2-amine (2 mmol, 0.283 g) and 2-bromoacetophenone (2 mmol, 0.498 g) in THF (10 ml) at 338 K catalyzed by iodine. m.p. 559–561 K. The single crystals suitable for X-ray diffraction were obtained by slow evaporation of an ethanol solution of (I).

### Refinement

H-atoms were included at geometrically idealized positions and refined in riding-model approximation with the following constraints: C—H distances were set to 0.95 Å and *U*_iso_(H) = 1.2*U*_eq_(C).

### Crystal data

**Table:**

C_25_H_15_ClN_2_O_2_	*Z* = 2
*M**_r_* = 410.84	*F*(000) = 424
Triclinic, *P*1	*D*_x_ = 1.443 Mg m^−^^3^
Hall symbol: -P 1	Melting point = 559–561 K
*a* = 9.1390 (12) Å	Mo *K*α radiation, λ = 0.71070 Å
*b* = 9.5350 (11) Å	Cell parameters from 3276 reflections
*c* = 11.9668 (17) Å	θ = 1.9–27.9°
α = 108.182 (4)°	µ = 0.23 mm^−^^1^
β = 105.366 (4)°	*T* = 113 K
γ = 92.739 (3)°	Block, yellow
*V* = 945.6 (2) Å^3^	0.34 × 0.32 × 0.22 mm

### Data collection

**Table:**

Rigaku Saturn diffractometer	4467 independent reflections
Radiation source: rotating anode	3779 reflections with *I* > 2σ(*I*)
confocal	*R*_int_ = 0.034
Detector resolution: 14.63 pixels mm^-1^	θ_max_ = 27.9°, θ_min_ = 2.3°
ω scans	*h* = −12→12
Absorption correction: multi-scan (*CrystalClear*; Rigaku, 1999)	*k* = −12→12
*T*_min_ = 0.926, *T*_max_ = 0.952	*l* = −15→15
11798 measured reflections	

### Refinement

**Table:**

Refinement on *F*^2^	Primary atom site location: structure-invariant direct methods
Least-squares matrix: full	Secondary atom site location: difference Fourier map
*R*[*F*^2^ > 2σ(*F*^2^)] = 0.049	Hydrogen site location: inferred from neighbouring sites
*wR*(*F*^2^) = 0.129	H-atom parameters constrained
*S* = 1.06	*w* = 1/[σ^2^(*F*_o_^2^) + (0.0619*P*)^2^ + 0.2446*P*] where *P* = (*F*_o_^2^ + 2*F*_c_^2^)/3
4467 reflections	(Δ/σ)_max_ = 0.001
271 parameters	Δρ_max_ = 0.36 e Å^−^^3^
0 restraints	Δρ_min_ = −0.47 e Å^−^^3^

### Special details

**Table:**

Geometry. All e.s.d.'s (except the e.s.d. in the dihedral angle between two l.s. planes) are estimated using the full covariance matrix. The cell e.s.d.'s are taken into account individually in the estimation of e.s.d.'s in distances, angles and torsion angles; correlations between e.s.d.'s in cell parameters are only used when they are defined by crystal symmetry. An approximate (isotropic) treatment of cell e.s.d.'s is used for estimating e.s.d.'s involving l.s. planes.
Refinement. Refinement of *F*^2^ against ALL reflections. The weighted *R*-factor *wR* and goodness of fit *S* are based on *F*^2^, conventional *R*-factors *R* are based on *F*, with *F* set to zero for negative *F*^2^. The threshold expression of *F*^2^ > σ(*F*^2^) is used only for calculating *R*-factors(gt) *etc*. and is not relevant to the choice of reflections for refinement. *R*-factors based on *F*^2^ are statistically about twice as large as those based on *F*, and *R*- factors based on ALL data will be even larger.

### Fractional atomic coordinates and isotropic or equivalent isotropic displacement parameters (Å^2^)

**Table:**

	*x*	*y*	*z*	*U*_iso_*/*U*_eq_	
Cl1	−0.44740 (5)	1.45046 (5)	0.65255 (4)	0.03265 (15)	
O1	0.11873 (18)	0.19922 (14)	0.55020 (13)	0.0390 (4)	
O2	0.20781 (16)	0.29045 (15)	0.43402 (12)	0.0373 (3)	
N1	0.09795 (15)	1.16854 (15)	0.97919 (12)	0.0190 (3)	
N2	0.16218 (16)	0.30287 (16)	0.52288 (13)	0.0237 (3)	
C1	0.03104 (17)	1.11236 (17)	0.85802 (15)	0.0180 (3)	
C2	0.05962 (18)	0.97511 (17)	0.78672 (15)	0.0197 (3)	
H2	0.0085	0.9370	0.7008	0.024*	
C3	0.16056 (18)	0.89462 (17)	0.83916 (14)	0.0178 (3)	
C4	0.24330 (17)	0.95634 (17)	0.96652 (15)	0.0181 (3)	
C5	0.20143 (17)	1.09302 (17)	1.03223 (15)	0.0184 (3)	
C6	0.26619 (18)	1.15733 (18)	1.16297 (15)	0.0212 (3)	
H6	0.2361	1.2475	1.2062	0.025*	
C7	0.36952 (19)	1.09148 (19)	1.22567 (15)	0.0228 (4)	
H7	0.4074	1.1337	1.3128	0.027*	
C8	0.42320 (18)	0.95928 (18)	1.16351 (15)	0.0208 (3)	
C9	0.36459 (18)	0.89220 (17)	1.03335 (15)	0.0193 (3)	
C10	0.43260 (18)	0.77063 (18)	0.97623 (16)	0.0221 (4)	
H10	0.4000	0.7273	0.8890	0.027*	
C11	0.54513 (19)	0.71333 (19)	1.04391 (17)	0.0253 (4)	
H11	0.5879	0.6308	1.0029	0.030*	
C12	0.59711 (19)	0.7757 (2)	1.17264 (17)	0.0265 (4)	
H12	0.6725	0.7342	1.2192	0.032*	
C13	0.53780 (19)	0.89731 (19)	1.23024 (16)	0.0244 (4)	
H13	0.5746	0.9411	1.3173	0.029*	
C14	−0.08359 (18)	1.19683 (17)	0.80328 (15)	0.0187 (3)	
C15	−0.17082 (19)	1.14279 (18)	0.67950 (15)	0.0224 (4)	
H15	−0.1543	1.0514	0.6265	0.027*	
C16	−0.28137 (19)	1.22157 (19)	0.63335 (16)	0.0237 (4)	
H16	−0.3407	1.1840	0.5492	0.028*	
C17	−0.30481 (18)	1.35505 (19)	0.71036 (16)	0.0222 (4)	
C18	−0.21770 (19)	1.41347 (19)	0.83272 (16)	0.0236 (4)	
H18	−0.2323	1.5067	0.8845	0.028*	
C19	−0.10882 (19)	1.33288 (18)	0.87788 (15)	0.0209 (3)	
H19	−0.0497	1.3713	0.9620	0.025*	
C20	0.16339 (17)	0.74070 (17)	0.75715 (15)	0.0184 (3)	
C21	0.21663 (19)	0.71865 (18)	0.65500 (15)	0.0216 (3)	
H21	0.2540	0.8023	0.6383	0.026*	
C22	0.21556 (18)	0.57585 (18)	0.57745 (15)	0.0214 (3)	
H22	0.2528	0.5604	0.5082	0.026*	
C23	0.15882 (18)	0.45621 (17)	0.60359 (14)	0.0193 (3)	
C24	0.09988 (19)	0.47391 (18)	0.70168 (15)	0.0221 (4)	
H24	0.0590	0.3899	0.7161	0.027*	
C25	0.10213 (19)	0.61767 (18)	0.77824 (15)	0.0215 (3)	
H25	0.0616	0.6326	0.8458	0.026*	

### Atomic displacement parameters (Å^2^)

**Table:**

	*U*^11^	*U*^22^	*U*^33^	*U*^12^	*U*^13^	*U*^23^
Cl1	0.0261 (2)	0.0316 (3)	0.0412 (3)	0.01053 (18)	0.0055 (2)	0.0162 (2)
O1	0.0580 (9)	0.0188 (6)	0.0367 (8)	0.0056 (6)	0.0139 (7)	0.0046 (6)
O2	0.0424 (8)	0.0319 (7)	0.0299 (7)	0.0032 (6)	0.0144 (6)	−0.0031 (6)
N1	0.0188 (7)	0.0175 (6)	0.0199 (7)	0.0015 (5)	0.0058 (6)	0.0054 (5)
N2	0.0208 (7)	0.0257 (7)	0.0182 (7)	0.0025 (6)	0.0012 (6)	0.0022 (6)
C1	0.0174 (7)	0.0159 (7)	0.0211 (8)	0.0008 (6)	0.0073 (6)	0.0057 (6)
C2	0.0201 (8)	0.0176 (7)	0.0193 (8)	0.0014 (6)	0.0053 (6)	0.0041 (6)
C3	0.0188 (7)	0.0149 (7)	0.0202 (8)	0.0015 (6)	0.0085 (6)	0.0042 (6)
C4	0.0175 (8)	0.0159 (7)	0.0216 (8)	0.0009 (6)	0.0073 (6)	0.0060 (6)
C5	0.0176 (7)	0.0166 (7)	0.0211 (8)	0.0013 (6)	0.0067 (6)	0.0056 (6)
C6	0.0229 (8)	0.0178 (7)	0.0206 (8)	0.0006 (6)	0.0070 (7)	0.0031 (6)
C7	0.0243 (8)	0.0227 (8)	0.0188 (8)	0.0001 (7)	0.0048 (7)	0.0053 (7)
C8	0.0192 (8)	0.0196 (8)	0.0249 (9)	−0.0003 (6)	0.0066 (7)	0.0094 (7)
C9	0.0176 (7)	0.0181 (7)	0.0235 (8)	0.0005 (6)	0.0075 (6)	0.0080 (7)
C10	0.0194 (8)	0.0216 (8)	0.0266 (9)	0.0029 (6)	0.0084 (7)	0.0082 (7)
C11	0.0209 (8)	0.0228 (8)	0.0354 (10)	0.0054 (7)	0.0107 (7)	0.0118 (8)
C12	0.0224 (8)	0.0281 (9)	0.0340 (10)	0.0061 (7)	0.0069 (8)	0.0182 (8)
C13	0.0235 (8)	0.0262 (9)	0.0232 (9)	0.0004 (7)	0.0033 (7)	0.0114 (7)
C14	0.0189 (8)	0.0169 (7)	0.0211 (8)	0.0016 (6)	0.0067 (6)	0.0072 (6)
C15	0.0251 (8)	0.0173 (8)	0.0231 (9)	0.0036 (6)	0.0065 (7)	0.0049 (7)
C16	0.0242 (8)	0.0232 (8)	0.0209 (9)	0.0017 (7)	0.0027 (7)	0.0072 (7)
C17	0.0182 (8)	0.0223 (8)	0.0289 (9)	0.0042 (6)	0.0074 (7)	0.0120 (7)
C18	0.0260 (9)	0.0197 (8)	0.0260 (9)	0.0055 (7)	0.0103 (7)	0.0062 (7)
C19	0.0227 (8)	0.0199 (8)	0.0188 (8)	0.0032 (6)	0.0061 (7)	0.0049 (7)
C20	0.0167 (7)	0.0165 (7)	0.0193 (8)	0.0041 (6)	0.0035 (6)	0.0037 (6)
C21	0.0239 (8)	0.0188 (8)	0.0221 (8)	0.0034 (6)	0.0067 (7)	0.0069 (7)
C22	0.0220 (8)	0.0243 (8)	0.0175 (8)	0.0057 (6)	0.0060 (7)	0.0058 (7)
C23	0.0183 (8)	0.0166 (7)	0.0175 (8)	0.0048 (6)	0.0019 (6)	0.0010 (6)
C24	0.0230 (8)	0.0175 (8)	0.0247 (9)	0.0024 (6)	0.0061 (7)	0.0064 (7)
C25	0.0240 (8)	0.0194 (8)	0.0216 (8)	0.0032 (6)	0.0097 (7)	0.0054 (7)

### Geometric parameters (Å, °)

**Table:**

Cl1—C17	1.7414 (17)	C11—H11	0.9500
O1—N2	1.2142 (19)	C12—C13	1.368 (2)
O2—N2	1.2165 (19)	C12—H12	0.9500
N1—C1	1.333 (2)	C13—H13	0.9500
N1—C5	1.359 (2)	C14—C19	1.396 (2)
N2—C23	1.487 (2)	C14—C15	1.398 (2)
C1—C2	1.401 (2)	C15—C16	1.388 (2)
C1—C14	1.490 (2)	C15—H15	0.9500
C2—C3	1.377 (2)	C16—C17	1.383 (2)
C2—H2	0.9500	C16—H16	0.9500
C3—C4	1.424 (2)	C17—C18	1.385 (2)
C3—C20	1.495 (2)	C18—C19	1.386 (2)
C4—C5	1.425 (2)	C18—H18	0.9500
C4—C9	1.464 (2)	C19—H19	0.9500
C5—C6	1.432 (2)	C20—C21	1.393 (2)
C6—C7	1.351 (2)	C20—C25	1.396 (2)
C6—H6	0.9500	C21—C22	1.386 (2)
C7—C8	1.432 (2)	C21—H21	0.9500
C7—H7	0.9500	C22—C23	1.386 (2)
C8—C13	1.412 (2)	C22—H22	0.9500
C8—C9	1.423 (2)	C23—C24	1.384 (2)
C9—C10	1.413 (2)	C24—C25	1.386 (2)
C10—C11	1.379 (2)	C24—H24	0.9500
C10—H10	0.9500	C25—H25	0.9500
C11—C12	1.402 (3)		
			
C1—N1—C5	118.45 (14)	C12—C13—C8	121.65 (16)
O1—N2—O2	124.78 (15)	C12—C13—H13	119.2
O1—N2—C23	117.66 (14)	C8—C13—H13	119.2
O2—N2—C23	117.56 (14)	C19—C14—C15	118.20 (15)
N1—C1—C2	121.44 (14)	C19—C14—C1	119.49 (14)
N1—C1—C14	117.00 (14)	C15—C14—C1	122.29 (14)
C2—C1—C14	121.43 (14)	C16—C15—C14	120.53 (15)
C3—C2—C1	121.03 (15)	C16—C15—H15	119.7
C3—C2—H2	119.5	C14—C15—H15	119.7
C1—C2—H2	119.5	C17—C16—C15	119.71 (15)
C2—C3—C4	119.17 (14)	C17—C16—H16	120.1
C2—C3—C20	115.55 (14)	C15—C16—H16	120.1
C4—C3—C20	125.06 (14)	C16—C17—C18	121.18 (15)
C3—C4—C5	115.47 (14)	C16—C17—Cl1	119.34 (13)
C3—C4—C9	125.96 (14)	C18—C17—Cl1	119.48 (13)
C5—C4—C9	118.57 (14)	C17—C18—C19	118.48 (15)
N1—C5—C4	124.14 (15)	C17—C18—H18	120.8
N1—C5—C6	115.65 (14)	C19—C18—H18	120.8
C4—C5—C6	120.19 (14)	C18—C19—C14	121.87 (15)
C7—C6—C5	120.86 (15)	C18—C19—H19	119.1
C7—C6—H6	119.6	C14—C19—H19	119.1
C5—C6—H6	119.6	C21—C20—C25	119.49 (15)
C6—C7—C8	121.35 (15)	C21—C20—C3	120.79 (14)
C6—C7—H7	119.3	C25—C20—C3	119.58 (14)
C8—C7—H7	119.3	C22—C21—C20	120.64 (15)
C13—C8—C9	119.58 (15)	C22—C21—H21	119.7
C13—C8—C7	120.33 (15)	C20—C21—H21	119.7
C9—C8—C7	120.06 (15)	C23—C22—C21	118.27 (15)
C10—C9—C8	117.29 (15)	C23—C22—H22	120.9
C10—C9—C4	124.07 (15)	C21—C22—H22	120.9
C8—C9—C4	118.58 (14)	C24—C23—C22	122.69 (15)
C11—C10—C9	121.62 (16)	C24—C23—N2	118.94 (15)
C11—C10—H10	119.2	C22—C23—N2	118.37 (14)
C9—C10—H10	119.2	C23—C24—C25	118.12 (15)
C10—C11—C12	120.63 (16)	C23—C24—H24	120.9
C10—C11—H11	119.7	C25—C24—H24	120.9
C12—C11—H11	119.7	C24—C25—C20	120.71 (15)
C13—C12—C11	119.08 (15)	C24—C25—H25	119.6
C13—C12—H12	120.5	C20—C25—H25	119.6
C11—C12—H12	120.5		
			
C5—N1—C1—C2	3.2 (2)	C9—C8—C13—C12	1.5 (2)
C5—N1—C1—C14	179.07 (13)	C7—C8—C13—C12	−176.50 (15)
N1—C1—C2—C3	−1.3 (2)	N1—C1—C14—C19	3.9 (2)
C14—C1—C2—C3	−177.01 (14)	C2—C1—C14—C19	179.74 (15)
C1—C2—C3—C4	−3.6 (2)	N1—C1—C14—C15	−174.37 (15)
C1—C2—C3—C20	171.36 (14)	C2—C1—C14—C15	1.5 (2)
C2—C3—C4—C5	6.1 (2)	C19—C14—C15—C16	−1.2 (2)
C20—C3—C4—C5	−168.31 (14)	C1—C14—C15—C16	177.03 (15)
C2—C3—C4—C9	−173.89 (14)	C14—C15—C16—C17	0.3 (3)
C20—C3—C4—C9	11.7 (3)	C15—C16—C17—C18	1.3 (3)
C1—N1—C5—C4	−0.2 (2)	C15—C16—C17—Cl1	−178.25 (13)
C1—N1—C5—C6	−178.50 (14)	C16—C17—C18—C19	−1.9 (3)
C3—C4—C5—N1	−4.4 (2)	Cl1—C17—C18—C19	177.61 (12)
C9—C4—C5—N1	175.56 (14)	C17—C18—C19—C14	1.0 (3)
C3—C4—C5—C6	173.80 (14)	C15—C14—C19—C18	0.6 (2)
C9—C4—C5—C6	−6.2 (2)	C1—C14—C19—C18	−177.75 (15)
N1—C5—C6—C7	179.58 (15)	C2—C3—C20—C21	66.7 (2)
C4—C5—C6—C7	1.2 (2)	C4—C3—C20—C21	−118.66 (18)
C5—C6—C7—C8	2.9 (3)	C2—C3—C20—C25	−108.78 (17)
C6—C7—C8—C13	176.32 (15)	C4—C3—C20—C25	65.8 (2)
C6—C7—C8—C9	−1.7 (2)	C25—C20—C21—C22	−2.9 (2)
C13—C8—C9—C10	−4.0 (2)	C3—C20—C21—C22	−178.39 (15)
C7—C8—C9—C10	174.05 (14)	C20—C21—C22—C23	0.7 (2)
C13—C8—C9—C4	178.58 (14)	C21—C22—C23—C24	1.7 (2)
C7—C8—C9—C4	−3.4 (2)	C21—C22—C23—N2	−178.06 (14)
C3—C4—C9—C10	10.0 (3)	O1—N2—C23—C24	−3.1 (2)
C5—C4—C9—C10	−170.04 (15)	O2—N2—C23—C24	176.60 (14)
C3—C4—C9—C8	−172.80 (14)	O1—N2—C23—C22	176.65 (15)
C5—C4—C9—C8	7.2 (2)	O2—N2—C23—C22	−3.6 (2)
C8—C9—C10—C11	3.6 (2)	C22—C23—C24—C25	−1.8 (2)
C4—C9—C10—C11	−179.09 (15)	N2—C23—C24—C25	177.99 (14)
C9—C10—C11—C12	−0.7 (3)	C23—C24—C25—C20	−0.5 (2)
C10—C11—C12—C13	−1.9 (3)	C21—C20—C25—C24	2.8 (2)
C11—C12—C13—C8	1.5 (3)	C3—C20—C25—C24	178.38 (15)

### Hydrogen-bond geometry (Å, °)

**Table:**

*D*—H···*A*	*D*—H	H···*A*	*D*···*A*	*D*—H···*A*
C24—H24···Cg^i^	0.95	2.72	3.510 (12)	142

Symmetry codes: (i) *x*, *y*−1, *z*.
